# Measuring the Cytochrome *c* Nitrite Reductase Activity—Practical Considerations on the Enzyme Assays

**DOI:** 10.1155/2010/634597

**Published:** 2010-06-22

**Authors:** Célia M. Silveira, Stéphane Besson, Isabel Moura, José J. G. Moura, M. Gabriela Almeida

**Affiliations:** ^1^REQUIMTE, Departamento de Química, CQFB, Faculdade de Ciências e Tecnologia, Universidade Nova de Lisboa, 2829-516 Caparica, Portugal; ^2^Faculdade de Ciências Médicas, Universidade Lusófona de Humanidades e Tecnologias, Campo Grande 376, 1749-024 Lisboa, Portugal; ^3^Escola Superior de Saúde Egas Moniz, Monte de Caparica, 2829-511 Caparica, Portugal

## Abstract

The cytochrome *c* nitrite reductase (ccNiR) from *Desulfovibrio desulfuricans* ATCC 27774 is able to reduce nitrite to ammonia in a six-electron transfer reaction. Although extensively characterized from the spectroscopic and structural points-of-view, some of its kinetic aspects are still under explored. In this work the kinetic behaviour of ccNiR has been evaluated in a systematic manner using two different spectrophotometric assays carried out in the presence of different redox mediators and a direct electrochemical approach. Solution assays have proved that the specific activity of ccNiR decreases with the reduction potential of the electronic carriers and ammonium is always the main product of nitrite reduction. The catalytic parameters were discussed on the basis of the mediator reducing power and also taking into account the location of their putative docking sites with ccNiR. Due to the fast kinetics of ccNiR, electron delivering from reduced electron donors is rate-limiting in all spectrophotometric assays, so the estimated kinetic constants are apparent only. Nevertheless, this limitation could be overcome by using a direct electrochemical approach which shows that the binding affinity for nitrite decreases whilst turnover increases with the reductive driving force.

## 1. Introduction

Cytochrome *c* nitrite reductase (ccNiR) is involved in the pathway called dissimilatory nitrate reduction to ammonia, thereby playing an important part in the biogeochemical nitrogen cycle. This enzyme catalyzes the six-electron reduction of nitrite (NO_2_^−^) to ammonia (NH_4_^+^) (1) and its existence has been demonstrated in bacterial strains from almost every taxonomic branch. However, the best studied ccNiRs were isolated from proteobacteria that belong to subdivisions *γ* (ex: *Escherichia coli*) [1], *δ* (ex: *Desulfovibrio desulfuricans* ATCC 27774) [2], or *ε* (ex: *W. succinogenes* or *Sulfospirillum deleyianum*) [3, 4]. The reaction mechanism has been particularly well studied in *Wolinella succinogenes. *The proposed model was based on the crystallographic observation of reaction intermediates and suggests that nitrite is reduced to ammonia without the release of nitric oxide, hydroxylamine, or any other intermediate [5]:

(1) $${{\text{NO}}_{2}}^{-}+{8\text{H}}^{+}+{6\text{e}}^{-}⟷{{\text{NH}}_{4}}^{+}+2\;{\text{H}}_{2}\text{O}$$

In addition to nitrite, some ccNiRs were shown to reduce other substrates like nitric oxide, hydroxylamine, o-methylhydroxylamine [6], or sulphite [7, 8].

The physiological form of the enzyme is believed to be a double trimer of 2 NrfA and 1 NrfH subunits [9]. The catalytic subunit NrfA is a periplasmic membrane-associated pentaheme cytochrome *c* where the short distances between hemes allow a fast and efficient electron transfer. The active site has been reported as an unusual lysine-coordinated high-spin heme [4, 10]. NrfH is a small membrane-bound cytochrome comprising four *c*-type heme groups and it serves a double purpose. On one hand, it anchors the catalytic subunits to the membrane: in *D. desulfuricans*, NrfH and NrfA form a strong complex that is only completely dissociated in the presence of SDS [11]. On the other hand, it serves as a quinol oxidase, transferring electrons from the quinone pool to the catalytic subunits [11, 12].

Kinetic studies of ccNiR have been largely based on classical spectrophotometric methods using methyl viologen (MV) as an electron source [1, 2, 6–8, 13, 14]. Although these protocols provide a satisfactory turnover, the investigation of the enzyme behaviour requires a more systematic study. The use of a variety of artificial electron donors, with different redox potentials or ionic charges, might shed light on electron entry points and donor-protein interactions and provide rigorous criteria to establish new reliable protocols. However, practical details on the experimental conditions (mediator concentration should not be limiting, subtraction of noncatalytic reactions, etc.) are rarely taken into account. Moreover, the list of available redox mediators is not long: besides being suitable electron carriers to the enzyme, they must also have chromophore groups that enable the reaction monitoring by UV-Vis spectrophotometry.

Searching in another direction, Protein Film Voltammetry is a promising alternative to the conventional methods used in kinetic studies. In this technique, an extremely small amount of a redox protein is adsorbed onto a solid electrode as an electroactive film. By applying a driving force (potential difference) the protein molecules exchange electrons directly with the electrode surface and the resultant current flow is recorded as a function of the electrode potential. In the case of an enzyme, and in the presence of its substrate, the catalytic process results in a steady-state electron flow whose detection allows instantaneous measurement of turnover rates. The electrode itself works as the redox partner of the enzyme; as so, no electron shuttle species or cosubstrates are mediating the process and no inhibition or interference reactions are expected [14]. Pyrolytic graphite (PG) electrodes have shown to be very suitable for the direct electrochemical study of redox proteins. Nonetheless, following immobilization on the electrode surface, enzymes may retain or not their catalytic properties [15–18].

**Figure 1:**
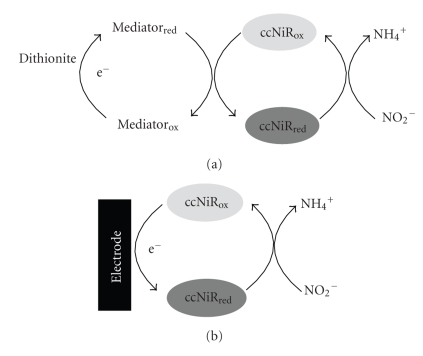
Reaction schemes for solution (a) and electrochemical (b) assays of ccNiR activity.

The aim of our work was to compare and discuss different experimental approaches to assess nitrite reductase activity, thereby contributing to the development of an efficient method, applicable in a broad range of experimental conditions. The kinetic behaviour of ccNiR was studied by two spectroscopic techniques and also by electrochemical assays, according to the experimental designs depicted in Figure 1.

For the spectroscopic techniques several redox mediators were used, namely, MV, Diquat (DQ), Phenosafranine (PS), Anthraquinone-2-sulphonate (AQS), and Indigo Carmine (IC).

This preliminary work could be quite relevant for the mechanistic study of nitrite reduction by ccNiR [19] as well as for paving the way towards the construction of new nitrite biosensors. In fact, several proposals of *D. desulfuricans* ccNiR- based nitrite biosensors were recently made, using different electronic mediators (MV and AQS) for enzyme activation [20–24]. In order to find the optimal conditions for biosensor operation and to make a consistent comparison between the analytical properties of the proposed systems, one should fully control the correspondent homogeneous enzymatic kinetics.

## 2. Experimental

### 2.1. Reagents

The multihemic nitrite reductase (ccNiR) (1.0 mg · mL^−1^) was purified from *D. desulfuricans* ATCC 27774 cells as previously described [11] and stored in 0.1 mol · L^−1^ phosphate buffer, pH 7.6, at −20°C. The protein concentration was determined with the Bicinchoninic Acid Protein Assay Kit (Sigma) using horse heart cytochrome *c* (Sigma) as standard.

1,1′-dimethyl-4,4′-bipyridinium dichloride (methyl viologen, MV), hydroxylamine, n-(1-naphtil)ethylenediamine, and phenol were purchased from Sigma. Sodium nitroprusside, 3,7-diamino-5-phenylphenazinium chloride (phenosafranine, PS), 5,5′-indigo sulfonic acid disodium (indigo carmine, IC), deiquat monohydrate (diquat, DQ), anthraquinone-2-sulphonate (AQS), sodium dithionite, potassium chloride, sodium nitrite, ammonium chloride, disodium hydrogen phosphate, sodium dihydrogen phosphate, and sulphanilamide were all from Merck. Hydrochloric acid was from Riedel-de-Haen. All chemicals were of analytical grade.

### 2.2. Enzymatic Measurements

#### 2.2.1. Discontinuous Assay

This assay involved two steps: the enzymatic conversion of nitrite to ammonia followed by the quantitative determination of both nitrite and ammonia present in the reaction mixture. For the enzymatic step, a 1 mL assay was prepared containing 0.2 M phosphate buffer pH 7.6, 0.5 mM sodium nitrite, 0.5 mM of mediator (0.25 mM in the case of PS, AQS, and IC), and appropriately diluted enzyme. In order to obtain comparable results, the mediator concentrations were levelled to provide the same reducing equivalents, since PS, AQS, and IC supply 2 electrons per molecule and the other mediators are one electron donors (when dithionite reduced). The reaction was initiated by the addition of 0.5 mM of sodium dithionite (in buffer solution), and after a particular incubation period (from 2 to 10 minutes) at 37°C, the reaction was stopped by oxidation of dithionite through vigorous stirring. The quantification of nitrite and ammonia was performed by the Griess [25] and indophenol blue [2, 26] methods, respectively. In control experiments performed without enzyme, using inactivated enzyme and without substrate, no ammonia formation (or nitrite consumption) was detected. One unit of enzyme activity is defined as the amount of enzyme that catalyzed the reduction of 1 *μ*mol of nitrite per minute.

#### 2.2.2. Continuous Assay

Kinetic data was obtained by monitoring the reoxidation of the mediators used as electron sources for enzyme turnover. The assay mixtures were prepared in a septum-stoppered quartz cell containing 0.16 mM of the mediators (except for PS-0.08 mM) and properly diluted ccNiR. Total cell volume was 2.5 mL completed with 0.2 M phosphate buffer, pH 7.6. The cell was purged with argon for 10 minutes before starting the reaction, while it was incubated at 37°C. Then, an appropriate volume of sodium dithionite was added into the cell with a syringe (typically *ca*. 20 *μ*L to achieve a final concentration in the cell of 0.16 mM, or 10 *μ*L when using PS) to reduce the mediators. The total volume could slightly vary from assay to assay due to some dithionite degradation following the solution deoxygenation step (the complete reduction of the mediators was assured by controlling the absorbance of their reduced forms).

The absorbance was measured for 30 seconds to establish a baseline (the rate of mediator non-enzymatic reoxidation, to be subtracted from nitrite reduction rates) and the reaction was subsequently started by the addition of nitrite stock solutions (1 and 10 mM). Kinetic measurements were made by following the rate of the change in light absorption of the mediators (604 nm for MV (*ε*_604 nm_ = 13.6 mM · cm^−1^), 460 nm for DQ (*ε*_460 nm_ = 2.7 mM · cm^−1^) and 540 nm for PS (*ε*_540 nm_ = 18.4 mM · cm^−1^)) in the presence of nitrite. Control assays performed in the absence of nitrite or enzyme showed little or no bleaching of the reduced mediators. Absorbance was measured with a Diode array spectrophotometer (Agilent Technologies 8453A UV-Vis). Kinetic parameters K_M_ and k_cat_ were determined using the software Graph Pad Prism 4.

#### 2.2.3. Electrochemical Assays

Chronoamperometry experiments (electrode potential was stepped and the current was measured) were performed with an Autolab electrochemical analyzer (PGSTAT12, Eco Chemie) under the control of GPES software (Eco Chemie). Electrode rotation was driven with an electrode rotator also controlled by GPES. The experiments were performed with a speed rotation of 600 rpm. A three-electrode cell configuration, composed of a silver/silver chloride reference electrode (Ag/AgCl), a platinum wire counter electrode, and a PG working electrode, was used. All potentials were quoted against NHE (+197 mV Ag/AgCl). The experiments were carried at 37°C in a single compartment cell containing 20 mL of supporting electrolyte (0.1 mol · L^−1^ phosphate buffer, pH 7.6). Solutions in the electrochemical cell were purged with argon before measurements and the argon atmosphere was maintained during the experiments by continuously flushing the cell. Protein films were prepared by depositing a 10 *μ*L drop of enzyme solution on the electrode surface. After 10 minutes, the electrode was washed with buffer and placed in the electrochemical cell. The protein surface coverage as estimated by the integration of the cyclic voltammogram of ccNiR obtained in the absence of nitrite was *ca*. 4 pmol · cm^−2^. The electrode response to nitrite was evaluated by successively adding small volumes of nitrite stock solutions (1, 10 and 100 mM), while continuously recording the activity. Control experiments showed no detectable faradaic current in the absence of ccNiR.

### 2.3. Molecular Docking Simulations

The atomic coordinates of *D. desulfuricans* ccNiR were obtained from the Brookhaven Protein Data Bank (entry 1OAH.pdb) [10]. Mediator structures were obtained from PubChem database (entries 15939 (MV), 6795 (DQ), 65733 (PS), 8551 (AQS), and 5284351 (IC)). The atomic coordinates of the mediators and ccNiR were used as input files for the docking algorithm PatchDock [27, 28], which creates potential complexes sorted according to shape complementarity criteria. The obtained complexes are sorted by a scoring function that considers both geometric fit and atomic desolvation energy. A root mean square deviation clustering of 4 Å is then applied to the complexes to discard redundant solutions. With this algorithm, the native result is typically found in the top 100 solutions and often among the top 10. The top 20 solutions obtained in this study were very similar to each other. Thus, to avoid presenting confusing pictures we decided to show only the first 5. Structure manipulation was done with the UCSF Chimera package [29].

## 3. Results and Discussion

### 3.1. Mediated Spectrophotometry

**Figure 2:**
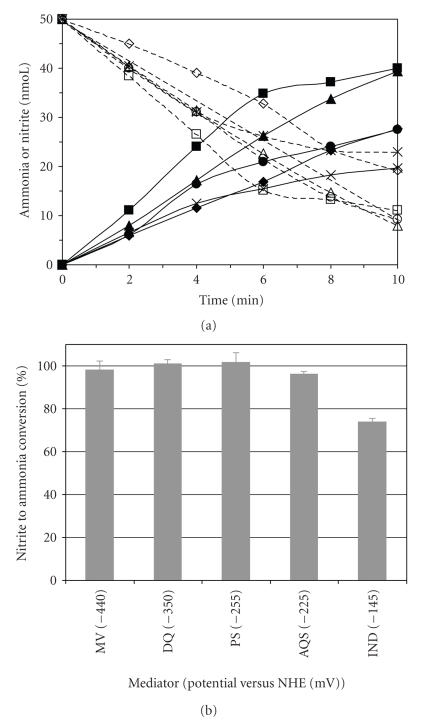
(a) Progression curves for ammonia (filled symbols) and nitrite (open symbols) for ccNiR reaction, in the presence of equivalent electron concentrations of dithionite reduced mediators at 37°C. MV (■□); DQ (♦*◊*); PS (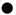 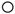); AQS (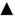 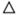) and IC (X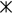). Enzyme concentration was 0.7 nM for MV, DQ, and PS assays, 7 nM for AQS, and 14 nM for IC. (b) Nitrite to ammonia conversion percentages in the presence of each mediator.

In the early stage of this study we investigated the influence of the electron donor in product formation. In particular, we intended to check if nitrite is directly converted to ammonia and no intermediates are accumulated during the course of the enzymatic reaction. We thus used a simple fixed-time method (single point), in which direct measurements of the reaction substrate and product were made [2]. In Figure 2(a) nitrite consumption and ammonia formation curves, traced in the presence of methyl viologen, diquat, phenosafranine, anthraquinone-2-sulphonate, and indigo carmine, are represented. Comparing the ammonia and nitrite contents we could confirm 100% conversion with all the mediators, except for indigo carmine (75%) as shown in Figure 2(b). The latter has the highest reduction potential tested (−145 mV); thus, we can speculate that the reaction is not complete and less reduced intermediates or products other than ammonium may be formed. No further studies were performed to corroborate this result, since efficient quantitation methods for reaction intermediates have yet to be established [7].

**Figure 3:**
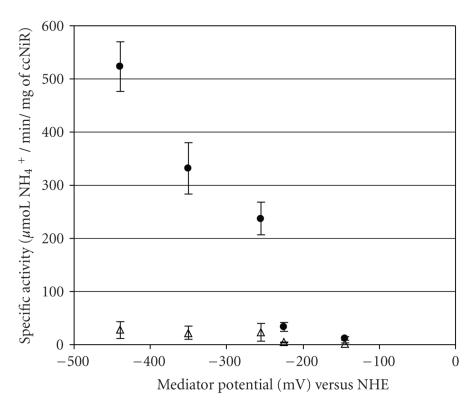
ccNiR specific activities determined with an equivalent electron concentration of the mediators and with a saturating nitrite (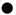) and hydroxylamine (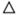)) concentration. Incubation time was 4 minutes, that is, within the period of ammonia production at a linear rate. Dithionite was used as reducing agent. All assays were performed at 37°C. Enzyme concentration was 0.7 nM for MV, DQ and PS assays, 7 nM for AQS, and 14 nM for IC.

The specific activity of ccNiR for nitrite and hydroxylamine was determined with all mediators, also using this discontinuous method. ccNiR hydroxylamine reducing activity was obtained by determining the amount of ammonia produced in the assay (hydroxylamine conversion percentages were not calculated, given that this substrate was not quantified). As shown in Figure 3, the activities for nitrite were at least 10 times greater than the activities for hydroxylamine, thus confirming nitrite as the specific substrate for ccNiR. Specific activity values were found to decrease with the potential of the electron carrier, indicating methyl viologen (the lowest potential mediator) as the most suitable electron donor for ccNiR. Activities are in the range found for ccNiRs from other organisms [1, 2, 6, 8, 31].

**Figure 4:**
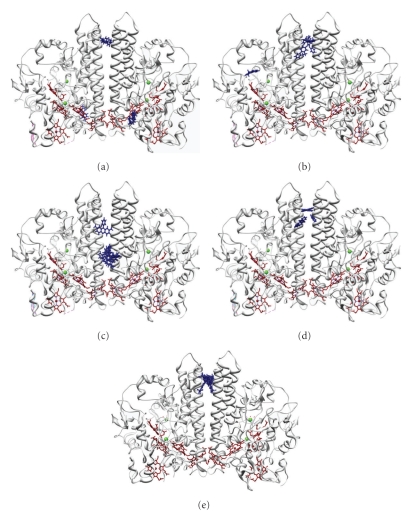
Interaction complexes of ccNiR with the mediators (a) MV, (b) DQ (c) PS, (d) AQS, and (e) IC. First five solutions are obtained with the molecular docking algorithm PatchDock. Heme groups are depicted in black and mediators are represented in ball and stick form.

Molecular docking studies were performed to evaluate the interaction between ccNiR and the redox mediators (Figure 4). From the best five solutions obtained with the molecular docking algorithm PatchDock [27, 28] we could validate MV as a convenient mediator for ccNiR reduction, since it interacts with a region closer to the electron transfer heme groups of the protein. In contrast, the highest potential mediators IC and AQS, which provided the lowest specific activities for nitrite reduction, interact with the enzyme in the interface between the two catalytic subunits, distant from any electron transfer hemes. Most of the top five solutions found for PS and DQ were also located in regions distant from the heme clusters, with the exception of DQ that has one putative docking site close to the catalytic heme.

The kinetic parameters for nitrite reductases are usually determined using the spectroscopic continuous method assay, in which the rate of reoxidation of a viologen cosubstrate gives an indirect measurement of enzyme activity [1, 6, 8, 13]. Although time-consuming, this type of method offers advantages relatively to the discontinuous assay applied above, since it allows direct tracking of the progress curve of the reaction, therefore making it relatively easy to estimate initial rates, spot any deviations from the initial linear phase of the reaction, and detect anomalous behaviours [32]. For this study, only the three lowest potential mediators (MV, DQ, and PS) were selected. The initial rates of ccNiR reaction were calculated from the changes in absorbance over time. In other words, the initial rate of mediator reoxidation, upon nitrite injection, was calculated by taking the first-derivative of the first part of the progression curve, thus considering a *pseudo*-1st-order reaction. In some cases the initial linear portion of the assay was sufficiently prolonged to allow the calculation of the initial rate reaction just by plotting a tangent to the first part of the assay curve, therefore considering a zero-order reaction rate.

**Figure 5:**
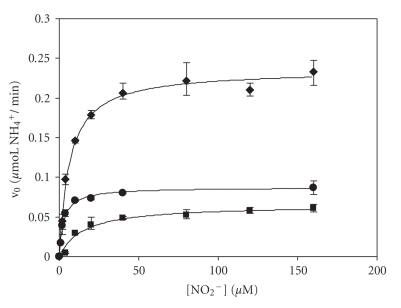
Enzyme activities determined using equivalent electron concentrations (0.16 mM) of MV (■), DQ (♦), PS (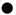), at 37°C, over increasing nitrite concentrations. The solid lines represent the simulations to the Michaelis-Menten equation. Enzyme concentration was 0.6 nM for MV, 1.5 nM for DQ, and 7.4 nM for PS assays.

An important consideration that must be drawn from the obtained results is that the reactions are not taking place at saturating mediator levels. The initial reaction velocities measured over a range of concentrations of MV, DQ, and PS, within the normal parameters used in spectroscopic assays (absorbance values between 0.5 and 2) were influenced by the mediator concentration (results not shown). Electron delivering from reduced electron donors is probably rate-limiting in this type of assay. This is not surprising since the need of six electrons per substrate molecule associated to the high catalytic rates of ccNiR demands a great supply of reducing equivalents. Therefore, the amount of electron donor in the reaction vessel should be increased, but this involves experimental challenges. The concentration of coloured mediators, such as MV and DQ (reduced forms), is always limited by the validity range of the Lambert-Beer's law; the absorbance eventually reaches such high levels that the apparatus no longer responds linearly to the increasing concentrations. Problem solving could have been provided by the nonabsorbing reduced form of phenosafranine, but its low solubility constituted another obstacle. Without a perfect option, and with the aim of obtaining a set of comparable kinetic data, we decided to level the concentration of the reducing equivalents that each mediator supplies (*cf*. experimental section). The obtained results are shown in Figure 5. In the presence of varying substrate concentrations the nitrite reducing activity follows a Michaelis-Menten profile for all mediators.

The apparent K_M_ and turnover numbers were calculated from data fitting to the Michaelis-Menten equation (Table 1). Although kinetic parameters are in the same order of magnitude, there is no coherent variation of the catalytic constant with the potential. In particular, the electronic carrier that gave the highest k_cat_ value is diquat, which has an intermediate reduction potential. This indicates a clear influence of the mediator nature on the measured activities, determined by other aspects than the reduction potential. Perhaps the electron transfer rate from DQ to the protein is much faster, enabling higher turnovers. In fact, according to the docking studies, DQ may interact differently with ccNiR, having some chances to deliver electrons directly to the catalytic site (Figure 4). 

The high k_cat_ value for DQ was also unexpected since in the discontinuous assay, the highest specific activity was observed for MV. A possible explanation can come from the fact that anaerobic conditions were maintained in the continuous method, but not in the discontinuous. As a result, in the latter assay the concentration of the reduced species is not properly controlled, as it is in the continuous mode (e.g., incomplete diquat reduction). This brings our attention to the artefacts that can be generated by indirect enzymatic assays based on measurements of electronic carriers.

The binding affinity for nitrite decreased consistently with the reduction potential (K_M_ rise). Possibly, in a fully reduced state the protein may adopt a structural conformation that somewhat diminishes the affinity for nitrite.

### 3.2. Direct Electrochemistry

The alternative method for determining nitrite reductase activity relied on a heterogeneous electron transfer reaction using an electrochemical technique. Herein ccNiR was adsorbed on the surface of a PG electrode that delivered electrons directly to the enzyme, thus converting it to its active state (Figure 1(b)). Due to the high activity of multihemic nitrite reductases it is difficult to eliminate mass-transport limitations in these experiments [15, 33]. An electrode rotation speed of 600 rpm was used in this work, which corresponds to 95% of the limiting current (not affected by mass transport). This was considered as a sufficient reflection of the steady-state activity of ccNiR. In this way, we were also able to avoid turbulence and flow irreproducibility generated at high electrode rotation speeds, by eddy currents and vortices forming around the edges of the revolving electrode [34]. 

**Figure 6:**
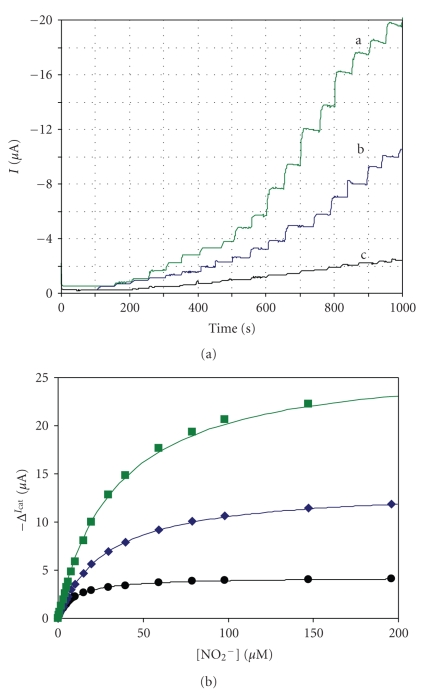
(a) Amperomograms of PG electrodes with immobilized ccNiR, in the presence of increasing amounts of nitrite, recorded at (a) −440 mV, (b) −350 mV, and (c) −255 mV. (b) Michaelis-Menten fittings to the calibration curves performed at −440 mV (■),−350 mV (♦), and −255 mV (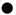). The assays were carried out at 37°C with an electrode rotation speed of 600 rpm. The solid lines represent the Michaelis-Menten simulations of enzyme kinetics.

The ccNiR activity for nitrite was determined by amperometric titration (Figure 6) using potentials equivalent to the ones provided by the redox mediators used in the spectroscopic assays (−440 mV for MV, −350 mV for DQ, and −255 mV for PS). In this way, in spite of varying the protein reduction level, the experimental conditions are fully comparable.

ccNiR films on the PG electrode exhibited turnovers equivalent to those measured in the solution assays (Table 1). Hence, the catalytic activity of the biofilm was greatly maintained. More importantly, the expected trend of increasing k_cat_ with the electrode reductive driving force was here observed. This should be related to the use of the same electron delivering system (PG electrode) at the three operating potentials, thus eliminating any variability coming from the use of different electronic mediators.

The Michaelis-Menten constants were slightly higher than those obtained in the solution assays. This may reflect a small effect of enzyme adsorption or mass transport. Yet, the binding affinity for nitrite also decreases with the reduction potential. Nonetheless, if one considers the k_cat_/K_M_ factor, the catalytic efficiency increases slightly with the reducing power regardless of the experimental approach.

## 4. Conclusions

The kinetic properties of ccNiR were studied using different methods. One of our goals was to study the influence of the electron donor of the reaction on the homogeneous kinetic parameters of ccNiR. As shown, below a sufficient reducing power (E < −250 mV), nitrite was always converted stoichiometrically to ammonia. However, the chemical structure of the electronic carrier may have a certain impact on ccNiR activity, most likely at the level of the mediator-protein interaction and the subsequent intermolecular electron transfer. Apparently, the enzyme has a greater affinity for diquat than for the others mediators. 

Methyl viologen was confirmed as the most suitable electron donor, since it provided the highest specific activity for nitrite. Moreover, it was the mediator that interacted more closely with the electron transfer hemes of ccNiR, as observed in the molecular docking studies.

We also conclude that we could not avoid the nonsaturating mediator conditions when using spectrophotometric techniques. As so, rate-limiting by electron donors is a pending issue and only apparent kinetic constants could be assessed. It should be stressed that this topic has not been considered in previous works. Thus, data presented in the literature should be evaluated carefully, taking into consideration the experimental conditions. Although the present study does not provide a way to determine real parameters describing the homogeneous kinetics, it indicates electrochemistry as a good alternative to techniques depending on chromophore mediators.

Our future plans should cover the study of ccNiR's interaction with substrates other than nitrite and hydroxylamine. A systematic study of enzyme inhibition, the influence of pH, ionic strength, and temperature should be very useful to understand the kinetic mechanism of ccNiRs and for developing a robust electrochemical biosensor.

## Figures and Tables

**Table 1:** Kinetic parameters for nitrite reduction catalysed by *D. desulfuricans *ccNiR obtained by the continuous spectrophotometric and the amperometric assays.

	Homogeneous			Electrochemical		
Mediator/ Potential (*vs* NHE)	K_M_^app^ (*μ*M)	k_cat_^app^ (s^−1^)	k_cat_/K_M_ (s^−1^ · *μ*M^−1^)	K_M_ (*μ*M)	k_cat_(s^−1^)*	k_cat_/K_M_ (s^−1^ · *μ*M^−1^)
Phenosafranine/−255 mV	2.7 ± 0.4	79 ± 3	29	9.2 ± 0.1	120 ± 1	13
Diquat/−350 mV	6.4 ± 0.6	1064 ± 23	166	28.1 ± 0.4	382 ± 2	14
Methyl viologen/−440 mV	15.0 ± 3.6	738 ± 45	49	33.3 ± 0.7	762 ± 4	23

*Turnover number was determined according to [30].
